# Shenqi Fuzheng Injection (SFI) Enhances IFN-*α* Inhibitory Effect on Hepatocellular Carcinoma Cells by Reducing VEGF Expression: Validation by Gene Silencing Technique

**DOI:** 10.1155/2019/8084109

**Published:** 2019-04-23

**Authors:** Xiaoheng Chen, Shuo Qi, Zhe Li, Bei He, Hui long Li, Jinxiang Fu, Sheng Huang, Lei Zhang, Xinai Li, Rui Hu, Lu Li, Tangshun Wang, Feng Xue, Xiang Gao, Xiaoguang Shi, Tao Zhang, Xin Wang, Junhui Wang, Zhiguo Ding

**Affiliations:** ^1^Department of Surgery, Dongzhimen Hospital of Beijing University of Chinese Medicine, Beijing, China; ^2^Department of Medical Service, The Third Clinical Medical College of Beijing University of Chinese Medicine, Beijing, China; ^3^Department of Physiology, Faculty of Medicine, University of Toronto, Toronto, Canada

## Abstract

Shenqi Fuzheng Injection (SFI) is a traditional Chinese medicine injection with anticancer properties and is mainly composed of ginseng and astragalus. Its efficacy has been confirmed in clinical trials, but the mechanism remains unclear. We investigated the effect of SFI on vascular endothelial growth factor (VEGF) gene expression in hepatocellular carcinoma (HCC) cells and identified its possible mechanism of synergistic effects when combined with the chemotherapeutic drug interferon (IFN-) *α*. An MTT assay was used to measure the inhibition effects of low-dose IFN-*α* (6000 IU) with or without SFI (0.5 g/L) on the HCC cell line MHCC97. VEGF-silenced MHCC97L-mir200 cell lines were prepared using lentiviral vectors and evaluated by real-time PCR to determine the inhibition effect. We examined MHCC97L-mir200 and MHCC97L cells by MTT assay, using IFN-*α* alone or in combination with SFI. The inhibition ratio of IFN-*α* (6000 IU) was -29.5%, while that for IFN-*α* (6000 IU) + SFI (0.5 g/L) was 17.0%, which was significantly higher than that for the IFN-*α* group (P < 0.01). The VEGF gene was silenced successfully in MHCC97-L cells. After interference of VEGF, the inhibition by SFI and IFN-*α* in MHCC97L-mir200 did not differ from that in MHCC97-L cells (P > 0.05). SFI can reduce the expression of VEGF in HCC, which can increase the efficacy of IFN-*α*, providing a theoretical basis for clinical application.

## 1. Introduction

Hepatocellular carcinoma (HCC) is a primary liver tumor and the most difficult human malignancy to treat. [1]. High rates of recurrence and metastasis after operation have become a bottleneck for improving the long-term efficacy, which is a key point in overcoming HCC. Interferon (IFN-) *α* is a chemotherapeutic drug that is widely used in the clinical treatment of HCC [2] and has various effects, such as resistance to viral infections, immune function, inhibition of cell proliferation, and apoptosis induction [3–6].

Angiogenesis is essential in the process of carcinogenesis to facilitate tumor progression and metastasis. Vascular endothelial growth factor (VEGF) is a well-characterized angiogenic factor known to stimulate angiogenesis within a tumor [7]. Studies have shown that increased expression of VEGF is an important factor in the recurrence and metastasis of HCC [8].

Previous studies showed that SFI can improve the survival time of HCC patients after operation, while also improving their quality of life [9–11].

The main components of Shenqi Fuzheng injection are ginseng and astragalus root. Ginseng has been traditionally used as a medicinal herb and food ingredient in dishes since ancient times in Asia and is now popular worldwide. Ginseng contains various bioactive components, including ginsenosides, phenolic compounds, polysaccharides, alkaloids, polyacetylenes, peptides, and fatty acids. The pharmacological effects of ginseng have been demonstrated in the nervous centralis, cardiovascular, and immune systems [12]. In addition, ginseng has been proposed to have chemopreventive effects on cancers of the lung and colon in animals and humans [13]. Among the constituents of ginseng, ginsenosides and polyacetylenes (the main components of ginseng) have been shown to decrease the malignant potential of liver cancer [14]. The dried root of* Astragalus membranaceus* (*Radix Astragali*) has a long history of medicinal use in traditional Chinese medicine for treating the common cold, diarrhea, fatigue, and anorexia as an immunomodulating agent in mixed herbal decoctions [15]. In contemporary pharmacotherapy,* R. astragali* has been used to ameliorate the side-effects of cytotoxic antineoplastic drugs [16]. Among its different constituents,* Astragalus* polysaccharides have been widely studied, particularly for their immunopotentiating properties, such as the stimulation of B cell proliferation and cytokine production in murine [17]. Additionally, clinical studies have shown that* Astragalus* polysaccharides can counteract the side-effects of chemotherapeutic drugs, including significantly attenuating myelosuppression in cancer patients [18].

In a previous study, we found that SFI can decrease various cytokines to reduce the invasiveness of cancer and enhance anticancer effects [19]. Therefore, this study of SFI focused on the expression of VEGF in the human HCC cell line MHCC97-L and its effects on VEGF expression of HCC cells.

## 2. Materials and Methods

### 2.1. Cell Culture

MHCC97-L cell lines were placed in 10% DMEM medium containing 10% fetal bovine serum (FBS) and penicillin (100 U/mL) and streptomycin (100 *μ*g/mL). Cell culture was performed at 37°C in humidified atmosphere containing 5% CO_2_. The cells were detached every 2–3 days with 0.25% trypsin/EDTA. The medium was exchanged after each passage. Cells were cultured for 24 h before initiation of the experiments.

### 2.2. Materials

Shenqi Fuzheng Injection (Li Beads group Limin Pharmaceutical, Shaoguan, China, S20110107), restructuring human interferon alpha-1B injection (Tri-prime gene limited, Beijing, China, S20010007), BCA protein concentration determination reagents box, predye protein markers, film closed liquid and ELC glow liquid (Thermo Fisher Scientific, Waltham, MA, USA); rat anti-people ICAM-1 monoclonal antibodies and rat anti-people STAT1 monoclonal antibodies (BD Biosciences, Franklin Lakes, NJ, USA); rabbit anti-people p-STAT1 monoclonal antibodies (Cell Signaling Technology, Danvers, MA, USA); anti-proliferating cell nuclear antigen monoclonal antibodies in rats (Abcam, Cambridge, UK), MTT, rat anti-*β*--actin monoclonal antibodies, horseradish peroxidase-conjugated goat anti-rat Mark II anti-rabbit and goat II antibodies (Santa Cruz Biotechnology, Dallas, TX, USA), Platinum HIFI Taq Polymerase, Trizol Reagent, 5×First-Strand buffer, 0.1 M DTT, SYBR Green I, Rnase out, oligo dT/Random primer, 10×PCR Buffer, Mg^2+^ (50 mM), Platinum Taq DNA Polymerase, 100 mM DNTPs (Invitrogen, Carlsbad, CA, USA), t-carrier, plenti6.3 MIR load, 293 cells, DH5 Alpha cells, ddwater (Heino bio Technology Ltd.), T4 Ligase (New England Biolabs, Ipswich, MA, USA); DMEM Medium, Hyclone, Logan, UT, USA); FBS (Biowest Company); 24 holes and 6-well cell culture plates (Corning), fluorescent Optical microscope (Olympus Corporation), fluorescence quantitative PCR instrument (Bio Rad).

### 2.3. Methods

#### 2.3.1. Cell Viability Assay of MHCC97L

To investigate whether the growth of the MHCC97L cell lines was inhibited and the extent of inhibition under SFI and IFN-*α*, a cell viability assay (MTT) was performed. Briefly, MHCC97L cells were seeded at a density of 40,000 cells/well in 0.2 mL in 96-well plates in the culture medium described above. After 24 h, SFI (0.5 g/L) 20 *μ*L, IFN-*α* (6000 IU) 20 *μ*L, or SFI (0.5 g/L) 10 *μ*L+IFN-*α* (6000 IU) were added separately to the wells. Cells treated with phosphate-buffered saline were used as a negative control. After 72 h incubation (5% CO_2_, 37°C), 15 *μ*L MTT solution was added to each well to reach a final concentration of 10 mg/mL. After further incubation for 2 h (5% CO_2_, 37°C), the cells were shaken for 5 min in a microvibration shaker at 4°C and the medium was removed. Formazan crystals present in the mitochondria of metabolically active cells were solubilized with 100 *μ*L of dimethyl sulfoxide, and the dye concentration was measured using an EL × 800 Microplate Reader (BioTek, Norcross, GA, USA) at a wavelength of 570 nm.

#### 2.3.2. Construction and Identification of Lentiviral Vector

MRNA interference sequences (Table 1) of the human VEGF gene (NCBI GenBank Gene ID: AF022375) were designed by using “BLOCK IT RNAi DESIGNER” (Invitrogen). Each complementary single strand (5 *μ*L) was mixed for annealing. The 4 oligo mixtures were heated at 95°C for 5 min, and then cooled for 20 min to form a double-chain oligo, which was connected to the linearized plenti6.3-MIR carrier to construct 2 miRNA lentiviral vector plasmids. The lentiviral vector was transformed into DH5*α* cells. Positive clones were screened by colony PCR with vector universal primers.

#### 2.3.3. Package of Lentivirus

Growing the 293T cells in log phase. After counting the cells, each Petri dish was inoculated with 6 × 10^6^ cells and incubated overnight at 37°C and 5% CO_2_. The culture medium was removed before transfection and 5 mL Opti-MEM medium was added. Next, 9 *μ*g packaging mixture and 3 *μ*g 1.5 mL lentiviral expression plasmid were added to Opti-MEM (37°C preheated) and mixed gently. After adding 36 *μ*L Lipofectamine 2000 to 1.5 mL Opti-MEM, the samples were mixed gently and placed for 5 min at 28°C. The plasmid solution and Lipofectamine 2000 were mixed gently and incubated for 20 min at room temperature. Plasmid-liposome compound (3 mL) was carefully added to cells in the Petri dish, mixed gently, and incubated for 6 h at 37°C and 5% CO_2_, after which the culture was replaced with DMEM containing 10% FBS. The supernatant was collected after 48 h cell culture and centrifuged for 10 min at 1509.3 xg (3000 rmp). Cells and debris were removed using a 0.45-*μ*m filter. The stock solution of virus was separated in an ultracentrifuge at 50,000 ×*g* for 2 h, the supernatant was removed, and the pellet was resuspended in 200 *μ*L DMEM and stored at −80°C.

#### 2.3.4. Determination of Lentivirus

293T cells were allowed to stand for one day before the measurements. Approximately 8000 cells in 100 *μ*L were added to each well. The stock solution of virus was diluted to different concentrations (each 50-*μ*L sample contained 1 × 10^−3^ to 1 × 10^-8 ^mL of lentivirus), and the original medium was removed from the 96-well plates. The lentiviral dilution medium was added at 50 *μ*L per well and mixed gently. Each dilution was tested in triplicate. After incubation for 48 h at 37°C and 5% CO_2_, we added 100 *μ*L of new medium to each well. Five days later, fluorescence signals were observed.

#### 2.3.5. Infection of MHCC97L and Real-Time PCR Detection of VEGF Expression

MHCC97L cells were cultured to the log phase and then infected for 6 h at a multiplicity of infection of 100. The culture medium was replaced with complete medium (without blasticidin) for the negative group and control group. After the cells were cultivated for 48 h, 5 *μ*g/mL blasticidin was added every 2–3 weeks (replaced fresh culture medium with antibiotics every 3–4 days). Cell death and the fluorescence ratio were observed by fluorescence microscopy. Cells were cultured until the cell fluorescence ratio reached above 90%.

After infecting MHCC97L cells for 48 h, we extracted cell RNA according to the Trizol cleavage fluid instructions and conducted RT-PCR reaction following the reverse transcription kit instructions. The primer sequences were as follows: VEGF upstream primer 5′-ACTGCCATCCAATCGAGACCC-3′, downstream primer 5′-TGAGGTTTGATCCGCATAATC-3′, *β*-Actin upstream primer 5′-ACTCTTCCAGCCTTCCTTCC-3′, downstream primer 5′-GTACTTGCGCTCAGGAGGAG-3′. The reaction system was formulated according to real-time PCR kit and amplified under the following conditions: predenaturation at 95°C for 120 s, 95°C for 10 s, 60°C for 30 s, and 70°C for 45 s for 40 cycles, and the real-time fluorescence signal was detected. MHCC97-blank group cells were used as a control, and the relative quantification was calculated by using the 2^−△△CT^ (CT means cycle threshold) method.

#### 2.3.6. Cell Viability Assay of MHCC97L-mir200

The cell viability assay (MTT) was performed again with VEGF gene silencing in MHCC97L cells (MHCC97L-mir200) to investigate the difference in the inhibition rate from normal MHCC97L cells. MHCC97L cells were seeded into 96-well plates at a density of 40,000 cells/well in 0.2 mL of the above-specified culture medium. After 24 h, SFI (0.5 g/L) 20 *μ*L, IFN-*α* (6000 IU) 20 *μ*L, or SFI (0.5 g/L) 10 *μ*L+IFN-*α* (6000 IU) were separately added to the wells. As a negative control, some cells were treated with phosphate-buffered saline. After 72 h incubation (5% CO_2_, 37°C), 15 *μ*L MTT solution was added to each well to reach a final concentration of 10 mg/mL. After further incubation for 2 h (5% CO_2_, 37°C) the cells were shaken for 5 min on a microvibration Shaker at 4°C and the medium was removed. Formazan crystals present in the mitochondria of metabolically active cells were solubilized with 100 *μ*L of dimethyl sulfoxide, and the dye concentration was measured by using an EL × 800 Microplate Reader at a wavelength of 570 nm.

## 3. Results

### 3.1. SFI Increases the Inhibitory Effect of IFN-*α* on MHCC97L Cells

**Figure 1:**
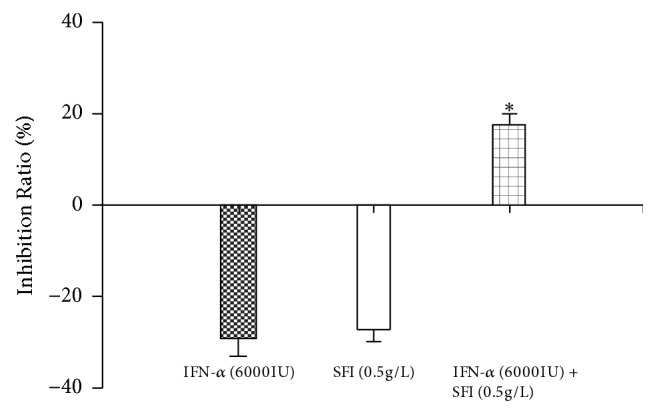
Inhibition ratio of SFI and INF-*α* on MHCC97L cells. *∗*P < 0.01, compared with IFN-*α* and SFI control group, means ± SEM, n = 5.

In the analysis of the inhibition ratio of MHCC97L cells with a low dose of IFN-*α* (6000 IU), SFI (0.5 g/L), and IFN-*α* (6000 IU) + SFI (0.5 g/L), the results showed that the inhibition ratio of a low dose of IFN-*α* (6000 IU) was -29.5%, while the value for SFI (0.5 g/L) was -27.4%, and IFN-*α* (6000 IU) + SFI (0.5 g/L) was 17.0%, which was significantly higher than that for the IFN-*α* (6000 IU) and the SFI (0.5 g/L) group (P < 0.01, compared to IFN-*α* and the SFI control group) (Figure 1).

### 3.2. SFI Decreases the Expression of VEGF on MHCC97L Cells

**Figure 2:**
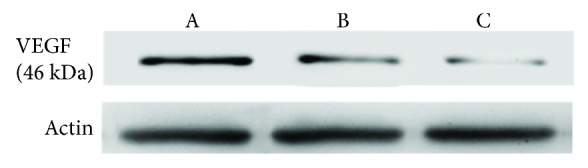
VEGF protein expression with SFI and INF-*α* treatment on MHCC97L cells. A. IFN-*α* (6000 IU); B. SFI (0.5 g/L); C. IFN-*α* (6000 IU) + SFI (0.5 g/L).

Western blot was used to analyze the VEGF expression of MHCC97L cells with a low dose of IFN-*α* (6000 IU), SFI (0.5 g/L), and IFN-*α* (6000 IU) + SFI (0.5 g/L). The results showed that the VEGF protein expression of a low dose of IFN-*α* (6000 IU) was higher than that for SFI (0.5 g/L) and IFN-*α* (6000 IU) + SFI (0.5 g/L) group (Figure 2).

### 3.3. Sequencing of Oligo Connected to plenti6.3-MIR

**Figure 3:**
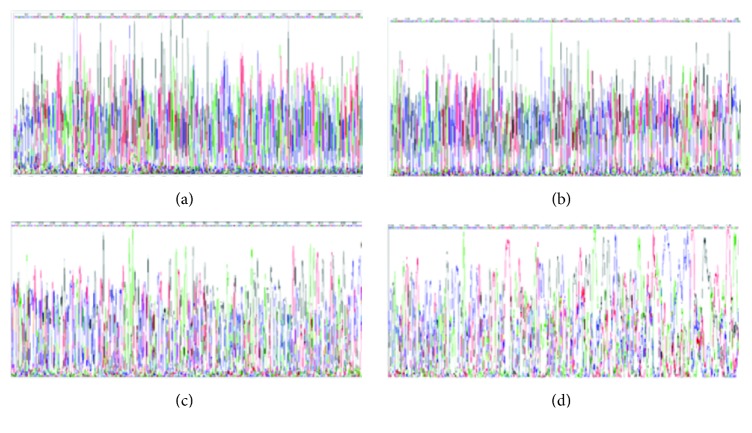
Sequencing results of oligo connection into the plenti6.3-MIR. (a) 0–300 bp; (b) 300–600 bp; (c) 600–900 bp; 900–1200 bp.

We determined the miRNA oligo sequences by sequencing to confirm that the oligo had been correctly connected to plenti6.3-MIR. The sequence was consistent with the expected result (Figure 3).

### 3.4. Lentiviral Packaging and Infection Succeeded

**Figure 4:**
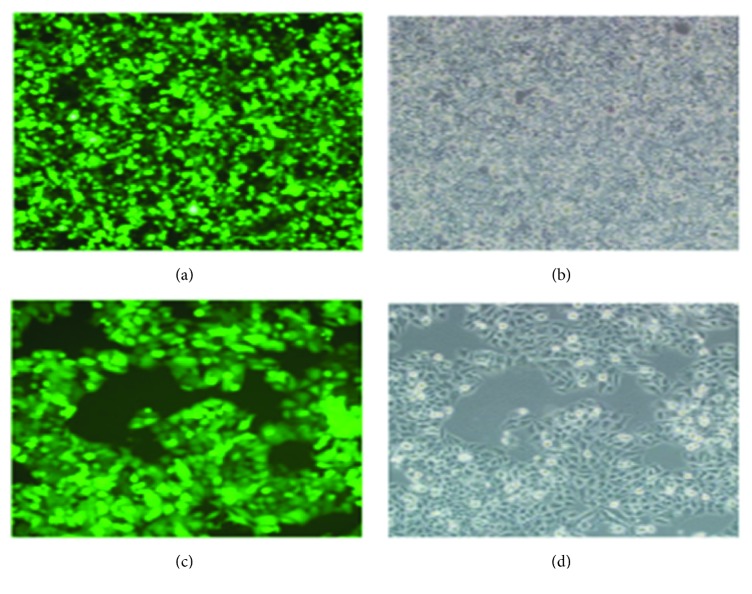
Effect of lentiviral infection after RNAi under the fluorescence microscope (×100). (a) Fluorescence at 24 h (b) visible light at 24 h. (c) Fluorescence at 48 h (d) visible light at 48 h.

Twenty-four hours after the plenti6.3-MIG-200 transfection experiment, similar findings were noted under both fluorescence and visible light (Figures 4(a) and 4(b)). Most cells were found to emit fluorescence, and the RNAi lentiviral infection rate of MHCC97L cells was over 90% at 48 h (Figures 3(c) and 3(d)), indicating that the transfection was successful.

### 3.5. Real-Time PCR

Real-time PCR was conducted to detect VEGF expression, and PCR analysis showed that the interference efficiency of the VEGF gene was up to 72.2% in MHCC97L-mir200 (Table 2).

### 3.6. SFI Increases the Inhibitory Effect of IFN-*α* on MHCC97L-mir200 Cells

**Figure 5:**
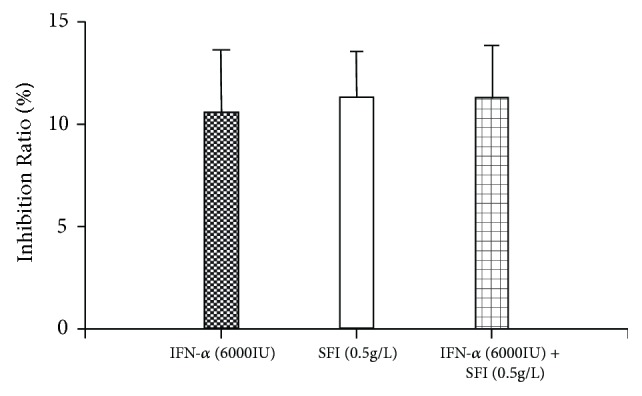
Inhibition ratio of SFI and INF-*α* on MHCC97L-mir200. P > 0.05, compared with IFN-*α* control group, SD + mean, n = 5.

After the VEGF gene was silenced by siRNA, we analyzed the inhibition ratio again. The MHCC97L-mir200 cells were treated with a low dose of IFN-*α* (6000 IU), SFI (0.5 g/L), and IFN-*α* (6000 IU) + SFI (0.5 g/L). The results showed that the inhibition ratio of a low dose of IFN-*α* (6000 IU) was 10.6%, SFI (0.5 g/L) was 11.5%, while that of IFN-*α* (6000 IU) + SFI (0.5 g/L) was 11.3%. There was no significant difference between the IFN-*α* (6000 IU) group, the SFI group, and combined group (P > 0.05, compared to the IFN-*α* and the SFI control group) (Figure 5).

## 4. Discussion

Many carcinomas have angiogenic mechanisms to induce tumor growth and disease progression [7]. VEGF signaling activates cellular pathways, which leads to the formation and branching of new tumor blood vessels, promotes rapid tumor growth, and facilitates metastatic potential [20]. Inhibiting the VEGF pathway may cause a rapid and sustained antitumor response [15]. SFI mainly contains* A. membranaceus* and ginseng. Formononetin is one of the constituents of* A. membranaceus*, and studies have shown that formononetin can downregulate the expression of VEGF [21].* Panax notoginseng* saponins were extracted from ginseng and analyzed. They were suggested to downregulate vascular endothelial growth factors in atherosclerotic vessels [22]. Therefore, SFI may also reduce the expression of VEGF, playing a synergistic antitumor role with IFN-*α*. First, the MTT assay results showed that SFI could indeed enhance the inhibitory effect of IFN-*α* in MHCC97L cells. To confirm that SFI has a key role in inhibiting the tumor by downregulating the expression of VEGF, we used siRNA to silence the VEGF gene in MHCC97L cells. To achieve this, we first constructed and packaged lentiviral vectors, which were used to infect MHCC97L cells and carried out real-time PCR detection of VEGF, which showed an interference efficiency of up to 72.2%. After the MHCC97L-mir200 cell lines were prepared, another MTT assay was conducted. The results showed that after the VEGF gene was silenced, there was no difference in the inhibition rate between using IFN-*α* alone and in combination with SFI. This also indicates that SFI exerts its effects by regulating the expression of the VEGF gene. Our results further confirmed the beneficial effects of SFI on the VEGF expression in consistence with previous report [23]. Although SFI is widely in China as an adjuvant drug for many cancers, such as lung and gastric cancers, the underlying mechanism is barely known. Regulating immunity in cancer patients with chemotherapy is the popular theory about the compound [24], our study here expanded the understanding to another important signaling way by looking at the VEGF and angiogenesis. As shown here, the cotreatment of SFI and cytokines could synergistically boost the inhibitory effect of IFN-*α* on cancer cell lines. Our study may provide helpful information for the application of SFI with immunomodulators in the treatment of cancers.

## 5. Conclusion

In summary, SFI can downregulate VEGF expression in HCC cells in a synergistic manner with IFN-*α*. VEGF is an important cytokine for tumor recurrence and development in a variety of solid tumors [25–28], providing a theoretical basis for clinical chemotherapy of carcinomas. SFI is a compound of natural drugs that has already been listed in China, but its mechanism is complex and not completely clear. Therefore, we will try to isolate the effective components in the future study and examine the antitumor effects of the monomer compositions.

## Figures and Tables

**Table 1:** miRNA oligo sequences (with negative control).

oligo	Oligo sequences 5′ to 3′
200-F	TGCTGTGAAGATGTACTCGATCTCATGTTTTGGCCACTGACTGACATGAGATCGTACATCTTCA

200-R	CCTGTGAAGATGTACGATCTCATGTCAGTCAGTGGCCAAAACATGAGATCGAGTACATCTTCAC

Negative-F	TGCTGAAATGTACTGCGCGTGGAGACGTTTTGGCCACTGACTGACGTCTCCACGCAGTACATTT

Negative-R	CCTGAAATGTACTGCGTGGAGACGTCAGTCAGTGGCCAAAACGTCTCCACGCGCAGTACATTTC

**Table 2:** PCR of VEGF expression and interference efficiency.

Sample	△CT	△△CT	2^−△△CT^	Interference efficiency
MHCC97L-mir200	10.652	1.847	0.278	0.722
MHCC97L-NEGA	8.412	-0.393	1.313	-0.313
MHCC97L-blank	8.805	0.000	1.000	0.000

## Data Availability

The data used to support the findings of this study are available from the corresponding author upon request.
